# Magnetic Properties of FeNi-Based Thin Film Materials with Different Additives

**DOI:** 10.3390/bios4030189

**Published:** 2014-07-04

**Authors:** Cai Liang, Chinthaka P. Gooneratne, Qing Xiao Wang, Yang Liu, Yogesh Gianchandani, Jurgen Kosel

**Affiliations:** 1 Computer, Electrical and Mathematical Sciences and Engineering, King Abdullah University of Science and Technology, 4700 Kaust, Thuwal 23955, Saudi Arabia; E-Mails: chinthaka.gooneratne@gmail.com (C.P.G.); jurgen.kosel@kaust.edu.sa (J.K.); 2 Core Laboratory and Major Facilities, King Abdullah University of Science and Technology, 4700 Kaust, Thuwal 23955, Saudi Arabia; E-Mails: qingxiao.wang@kaust.edu.sa (Q.X.W.); yang.liu@kaust.edu.sa (Y.L.); 3 Department of Electrical Engineering and Computer Science, University of Michigan, Ann Arbor, MI 48109, USA; E-Mail: yogesh@umich.edu

**Keywords:** magnetic sensor, magnetoelastic, magnetostriction, longitudinal vibration, magnetic thin films, magnetic materials

## Abstract

This paper presents a study of FeNi-based thin film materials deposited with Mo, Al and B using a co-sputtering process. The existence of soft magnetic properties in combination with strong magneto-mechanical coupling makes these materials attractive for sensor applications. Our findings show that FeNi deposited with Mo or Al yields magnetically soft materials and that depositing with B further increases the softness. The out-of-plane magnetic anisotropy of FeNi thin films is reduced by depositing with Al and completely removed by depositing with B. The effect of depositing with Mo is dependent on the Mo concentration. The coercivity of FeNiMo and FeNiAl is reduced to less than a half of that of FeNi, and a value as low as 40 A/m is obtained for FeNiB. The surfaces of the obtained FeNiMo, FeNiAl and FeNiB thin films reveal very different morphologies. The surface of FeNiMo shows nano-cracks, while the FeNiAl films show large clusters and fewer nano-cracks. When FeNi is deposited with B, a very smooth morphology is obtained. The crystal structure of FeNiMo strongly depends on the depositant concentration and changes into an amorphous structure at a higher Mo level. FeNiAl thin films remain polycrystalline, even at a very high concentration of Al, and FeNiB films are amorphous, even at a very low concentration of B.

## 1. Introduction

Magnetostrictive sensors have been investigated for various sensing applications, like biological, chemical, *etc*., for several years [1,2,3,4,5,6]. This type of sensor is made of magnetostrictive materials, e.g., Metglas 2826 MB, and structured in the fashion of either cantilevers or freestanding beams (particles). When an alternating magnetic field is applied to the sensor, it vibrates due to the magneto-mechanical coupling effect, and its fundamental resonant frequency in the longitudinal direction in air can be found approximately by [7]:

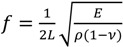
(1)
where *L*, *E*, *ν* and *ρ* are the sensor length, Young’s modules, Poisson’s ratio and density of the sensor material, respectively. When a uniformly distributed mass (Δ*m*) is added to the sensor without changing the physical properties of the sensor, except the density, the change in the resonant frequency of the sensor is [8],

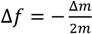
(2)
where *m* and *f* are the original mass and resonant frequency of the sensor before adding the mass, respectively. When target samples, e.g., biological molecules, bond to the sensor, the mass of the sensor increases, resulting in a frequency decrease. In order to increase the mass sensitivity, which is defined as the change in resonant frequency per unit of added mass,

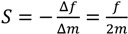
(3)
two effective approaches can be employed: (i) increasing the resonant frequency; and (ii) reducing the mass. The first can be achieved by reducing the length (see Equation (1)), whereas the second can be obtained by miniaturizing the overall size. Commercially available Metglas^TM^ 2826 MB ribbons with a composition of Fe_40_Ni_38_Mo_4_B_18_ offer soft magnetic properties and relatively large magneto-mechanical coupling constants and have been most efficiently employed as sensor platforms in such applications. However, the sensitivity of a freestanding beam sensor with a size of 1,000 × 200 µm that is made of Metglas 2826 MB with the standard thickness of 28 μm is only about 50 Hz/µg. If the thickness were to be reduced to 1 µm, the sensitivity would increase 28-times to about 1.4 Hz/pg. It is a challenge to reduce the thickness of Metglas 2826 MB ribbons to this value. Therefore, FeB and FeNiMoB thin films have been studied for microscale sensors, with promising results [8,9]. In [8], a FeB film was deposited by sputtering from Fe and B targets. In the resulting samples, some cracks were observed on the film surface, and the corrosion of the sensors occurred during the detection of biological targets. In addition, its coercivity was relatively high. In [9], a Metglas 2826MB ribbon was used as a sputtering target to deposit a FeNiMoB thin film and to fabricate microscale sensors. Since the target is made from Metglas ribbons, sputter deposition is limited to a very short time. In another study, FeNiMoB films were prepared using co-sputtering of FeNi, Mo and B targets [10]. Both the FeNiMoB films deposited in [9] and [10] showed magnetic properties close to Metglas 2826 MB; however, the deposition of FeNiMoB material requires a complex process—either co-sputtering from three targets or sputtering directly from a target made of Metglas 2826 MB ribbons. In any case, the challenges are to obtain the same composition and properties as those of Metglas 2826 MB and to accommodate three or more targets in a sputtering system, which are used for the co-sputtering process.

The goal of this work is to study the properties of FeNi-based thin films deposited with only one element out of Mo, Al and B to reduce the number of elements in the thin film material from four (as used in Metglas 2826 MB) to three, while achieving similar magnetic properties as Metglas 2826 MB and FeNiMoB thin films, as studied before in terms of coercivity and softness. As a result, the complexity of the fabrication process is reduced, and fewer cathodes are required in the sputtering system. The addition of Mo and B in Metglas improves the magnetic softness. In addition, the noble metal, Mo, is quite resistive to corrosion environments, which can improve the corrosion resistive properties of the FeNi material; whereas B can easily diffuse into Fe-Ni structures during thin film growing, due its small atomic size, which causes the formation of an amorphous alloy of FeNiB [11]. The introduction of Al to FeNi to form FeNiAl thin films is motivated by the relatively high magnetostrictive constant of FeAl materials. Earlier studies have shown that when a portion of Fe atoms are substituted by the nonmagnetic Al, its magnetostrictive constant increased to levels as high as 150 ppm for Al, reaching about 20 at% [12,13,14,15]. In contrast, the saturation magnetostriction of Fe_40_Ni_38_Mo_4_B_18_ ribbons is only about 12 ppm [16]. A high magnetostrictive coefficient is a prerequisite for a large change in the longitudinal dimension of a magnetostrictive sensor element, when it is subjected to a magnetic field, which is necessary for good sensor performance. This study primarily focuses on the properties of FeNi thin film materials with various additives towards better magnetic properties for biosensor applications.

## 2. Experiments

FeNi-based thin films were deposited on a Si substrate by using Ar plasma co-sputtering of an FeNi (50/50, at%) target and one of the depositing targets, Al, Mo or B, using a sputtering system from Equipment Support Corporation Ltd. All targets size were 75 mm in diameter and 3–5 mm in thickness. The purity of each element was 99.99%. The distance between the substrate and the target was 150 mm. The substrate holder was rotated at a constant speed of 20 rpm. All targets were sputter cleaned for 10 min with the shutter closed before each deposition, to remove any cross-contamination on the surfaces. The base pressure was set 3 × 10^–7^ Torr or lower, and the process pressure was set at 7 mTorr for all depositions. The deposition rate of each material was calibrated at different sputtering power levels. Table 1 lists the parameters used for thin film deposition with variable deposition time.

The microstructures of the thin film materials were characterized using a Bruker X-Ray Diffractometer (XRD) with Cu-*Kα* radiation, and an FEI Titan Super-Twin Transmission Electron Microscope (TEM). The compositions of FeNiMo and FeNiAl materials were characterized using a scanning electron microscope (SEM) equipped with an energy-dispersive X-ray spectroscopy (EDS) detector, and FeNiB was analyzed by X-ray photoelectron spectroscopy (XPS), a Kratos Axis Ultra DLD system from Kratos Analytical, due to the fact that the mass of B is light. The in-plane magnetic properties of the films were characterized using a vibrating sample magnetometer (VSM) system, Model 3900 from Princeton Measurements Corporation. The out-of-plane properties were characterized using an atomic force microscope/magnetic force microscope (AFM/MFM), Model 5400 from Agilent Technology. In order to obtain a FeNi film with the proper composition of additives, the individual deposition rates of FeNi, Mo, B and Al were obtained experimentally for different sputtering power, supported by calculation, to determine the sputtering power level, as reported in [10].

**Table 1:** Sputtering powers used for the deposition of thin films.

Film material	Sputtering power (W)			
	FeNi	Mo	Al	B
FeNi	200	-	-	-
FeNiMo-1	200	40	-	-
FeNiMo-2	200	45	-	-
FeNiMo-3	200	47	-	-
FeNiAl-1	200	-	50	-
FeNiAl-2	200	-	100	-
FeNiAl-3	200	-	150	-
FeNiB-1	225	-	-	150
FeNiB-2	225	-	-	175
FeNiB-3	225	-	-	200

## 3. Results and Discussion

### 3.1. Synthesis and Microstructure of Thin Film Materials

Table 2 lists the thickness and composition of the synthesized thin films and their magnetic properties.

**Table 2:** Thin films’ thickness, composition and magnetic properties.

Film material	Thickness (nm)	Composition (at%)				Hc (kA/m)	Mr (kA/m)	Ms (kA/m)
		FeNi	Mo	Al	B			
FeNi	420	100	0	0	0	6.08	0.66	4.40
FeNiMo-1	410	91.0	9.0	0	0	4.09	0.77	4.79
FeNiMo-2	420	89.4	10.6	0	0	3.65	0.86	4.64
FeNiMo-3	440	88.7	11.3	0	0	3.32	0.88	4.79
FeNiAl-1	410	84.4	0	15.6	0	4.00	1.64	5.34
FeNiAl-2	440	79.4	0	20.6	0	2.68	1.03	5.81
FeNiAl-3	450	73.1	0	26.9	0	2.10	0.62	2.96
FeNiB-1	540	90.8	0	0	9.2	0.16	0.51	7.80
FeNiB-2	550	89.2	0	0	10.8	0.04	0.50	9.38
FeNiB-3	580	87.4	0	0	12.6	0.08	0.49	10.54

Note: Mr and Ms are per unit volume of a cubic centimeter.

The EDS results show that the compositions of Mo and Al in FeNiMo and FeNiAl were in the range of 9.0 to 11.3 at% and 15.6 to 26.9 at%, respectively. In the case of FeNiB, the concentration of B was in the range of 9.2 to 12.6 at%, which was analyzed using XPS. The composition of Fe and Ni in the target is a 1:1 atomic ratio. In a previous paper [10], we showed that the actual composition of Fe and Ni in the film materials was slightly different from 1:1. In order to focus on the effects the additives have on FeNi films, only the combined amount of Fe and Ni is considered.

**Figure 1:**
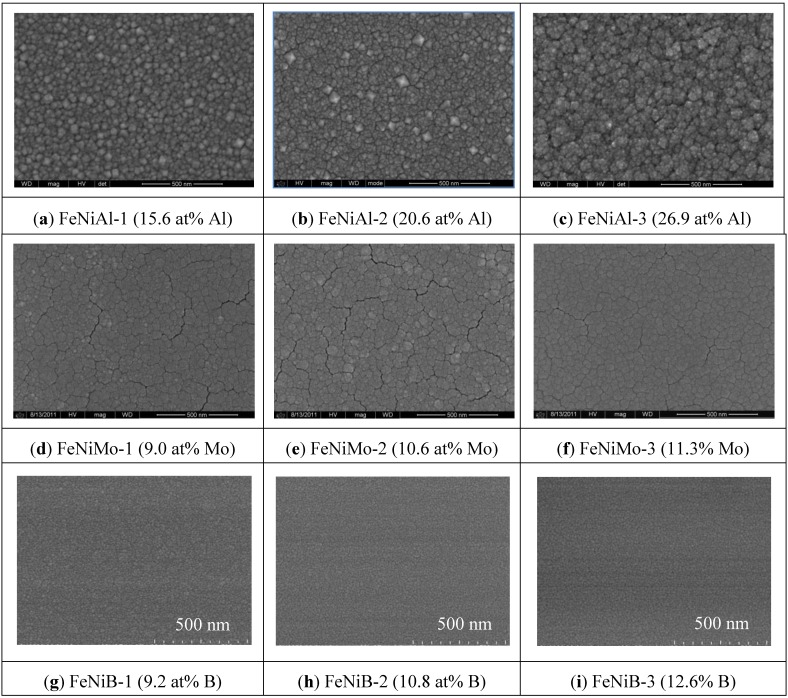
SEM images of the surface morphology evolutions of FeNiAl, FeNiMo and FeNiB with various Al, Mo and B contents.

FeNi thin films without any additive developed many cracks on the surface, as reported earlier [10]. The surface morphology of FeNi films with various concentrations of added elements is shown in Figure 1. Note that all of the SEM images are with 500 nm scale bars. FeNi deposited with Al resulted in a relatively coarse film with large clusters, as seen in Figure 1a–c. The surface morphology of FeNiMo became smooth, and the cracks became slightly less with the increase of Mo content, which can be seen in Figure 1d–f. Nano-granular clusters and a very smooth surface were obtained when the FeNi film was deposited with B, as seen in Figure 1g–i. No cracks were observed on the thin film surfaces when either Al or B was co-sputtered with FeNi.

**Figure 2:**
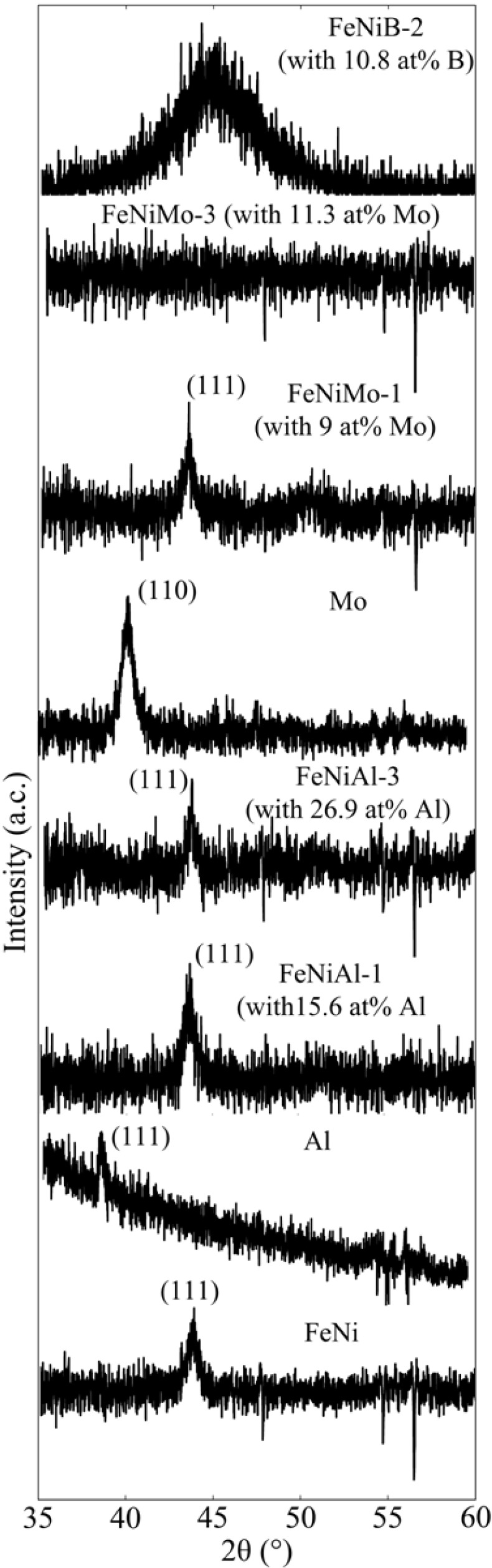
XRD spectrum of thin films, Al, Mo, FeNi, FeNiAl, FeNiMo and FeNiB. Mo exhibited a body-centered cubic (BCC) polycrystalline structure. Al and FeNi exhibited a face-centered cubic (FCC) polycrystalline.

Figure 2 shows the XRD spectra of Al, Mo, FeNi and FeNi thin films with various additive concentrations. The intensity of the XRD diffraction spectra is low, because the deposited film is thin. Due to the high diffraction intensity of the substrate (Si) at an angle of 2 theta of about 69°, there were no diffraction peaks near 75° and beyond. The XRD spectrum was recorded within the range of 2 theta between 35° and 60°. Al and FeNi thin films were characterized by a face-centered cubic (FCC) polycrystalline structure, and Mo was characterized by a body-centered cubic (BCC) structure. However, the sputter-deposited B was found to be amorphous (the spectrum is flat and not shown in Figure 2). Thin films of FeNi deposited with Mo were of an FCC polycrystalline structure at a low concentration of Mo (e.g., at 9.0 at%), and the structure gradually changed to amorphous with increasing concentration (e.g., 11.3 at%). However, this transition was not found for FeNi films deposited with either Al or B. FeNiAl films were also of an FCC polycrystalline structure for a concentration of Al in a range from 15.6 to 26.9 at%. The fact that the Al did not influence the crystal structure of FeNi is likely due to the similarity in the crystal structure of FeNi and Al films, which both exhibit an FCC structure at room temperature.

**Figure 3:**
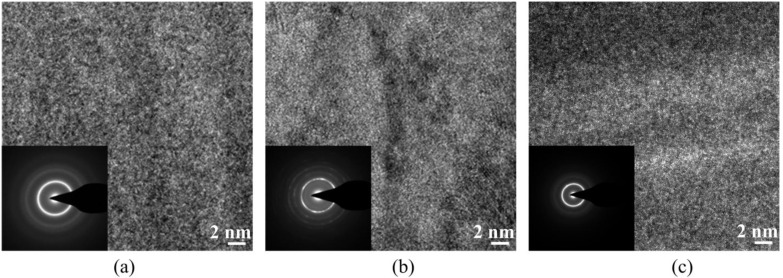
HRTEM images with selected area of diffraction (SAD) insets. (**a**) FeNiB thin film with 9.2 at% B, (**b**) FeNiAl thin film with 26.9 at% Al and (**c**) FeNiMo thin film with 11.3 at% Mo.

FeNi films deposited with various compositions of B exhibited an amorphous structure, even if the content was as low as 9.2 at% (Figure 2). A broad spectrum representing the (111) plane of FeNi is found in FeNiB thin films. This could be due to the small atomic size of B, which permits easy diffusion into the relatively large FCC structure of FeNi during the film nucleation and growth. As a result, a disordered structure of FeNi would be developed. High-resolution TEM images with selected area of diffraction (SAD) insets for FeNiB, FeNiAl and FeNiMo with 9.2 at% of B, 26.9 at% of Al and 11.3 at% of Mo, respectively, are shown in Figure 3. The FeNiB film is characterized by the absence of large grains (Figure 3a), while randomly distributed polycrystalline grains at various orientations are clearly seen in the case of the FeNiAl film (Figure 3b). The FeNiMo films exhibit an amorphous structure (Figure 3c). The SAD patterns indicate the amorphous structures of FeNiB and FeNiMo thin films and the polycrystalline structure of FeNiAl films, which confirms the results obtained using XRD.

### 3.2. In-Plane Magnetic Properties

The in-plane magnetic properties obtained for thin films of FeNi and FeNi deposited with different concentrations of Al, Mo and B are listed in Table 2. The coercivity (Hc) of FeNi is about 6 kA/m. Its coercivity decreases when deposited with any of the elements, and the higher the addition level of the element, the more the reduction. A significant reduction of Hc down to a value of 40 A/m is found when FeNi is deposited with B at a concentration of 10.8 at%. The remanence (Mr) of FeNi deposited with Mo increases with the Mo concentration in general, which has been reported before [10]. However, Mr decreases with an increase in the Al concentration when deposited with Al, and it does basically the same when deposited with B. A significant increase in the saturation magnetization (Ms) was observed when the FeNi was deposited with B, and the higher the B concentration, the more the increase. We assume that the changes of magnetic properties are mainly due to the change of the crystal structures when FeNi is deposited with different additives. The value of Hc decreases as the crystals size decreases in nano-crystalline-structured magnetic materials [17]. The surface morphology of the thin films also contributes to these changes. Smooth surfaces result in magnetically softer properties.

**Figure 4:**
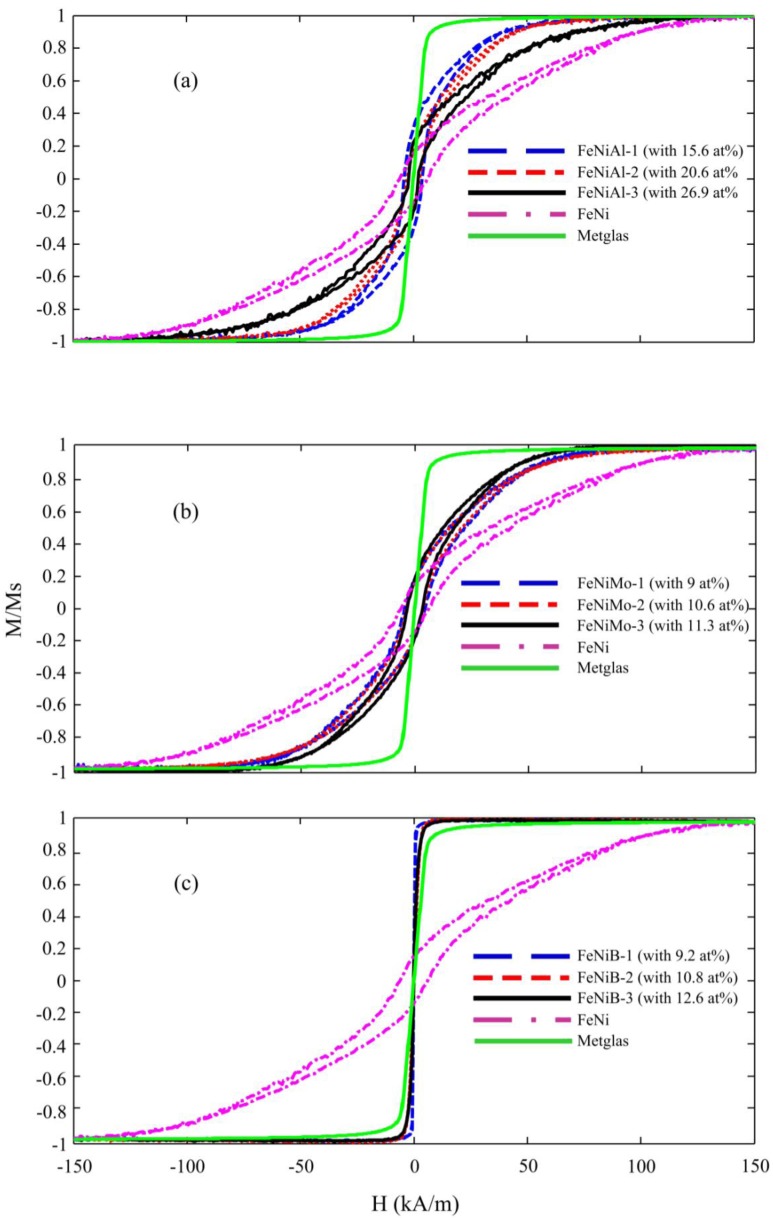
Magnetization loops for (**a**) FeNiAl, (**b**) FeNiMo and (**c**) FeNiB, at various concentrations of Al, Mo and B.

The in-plane magnetization behaviors of FeNi thin film materials with and without additives are plotted in Figure 4. A significant difference in the magnetization characteristics can be seen between films with different additives and different additive concentrations: (1) FeNi becomes magnetically softer when deposited with any of elements Mo, Al, or B; (2) The required magnetic fields to reach the saturation of magnetization for FeNiAl, FeNiMo and FeNiB are about 95 kA/m, 80 kA/m and 8 kA/m, respectively, which are considerably lower than the one required to saturate FeNi films without additives; and (3) in the lower range of the applied field strength, the magnetization change with the field strength (dM/dH), or the susceptibility, increases when FeNi is deposited with any of Mo, Al and B. The susceptibility slightly increases for the FeNiMo and FeNiAl, and a massive increase is obtained in the case of FeNiB. The three outstanding properties of the FeNiB films, a significant decrease in both coercivity and the saturation magnetization field and an increase in susceptibility, are due to the smooth, nano-granular clusters and amorphous structure of the films, which completely remove the anisotropy in the direction perpendicular to the film’s surface.

### 3.3. Out-of-Plane Magnetic Properties

**Figure 5:**
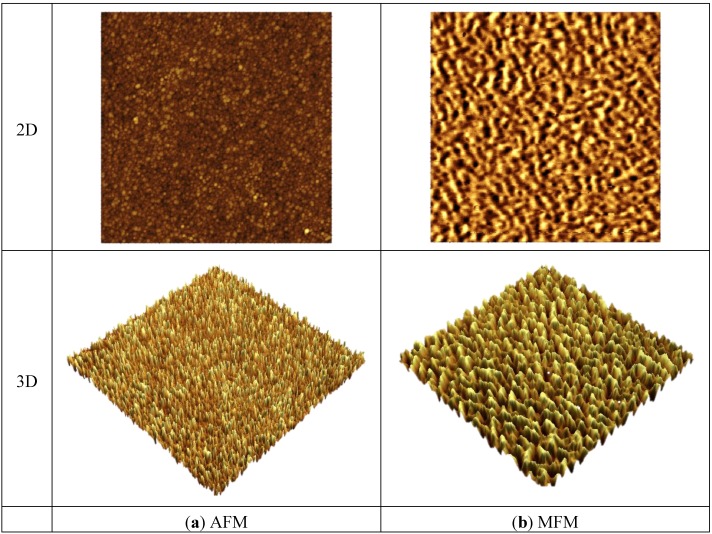
AFM and MFM images of FeNi thin film. (**a**) AFM images in 2D and 3D; (**b**) MFM images in 2D and 3D.

**Figure 6:**
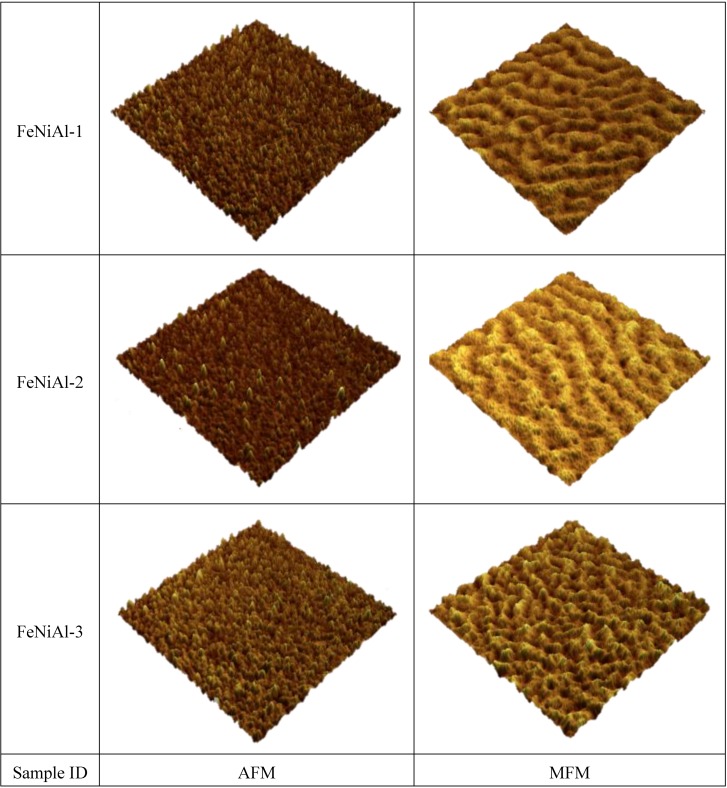
AFM and MFM images (size: 3 µm × 3 µm) of FeNiAl thin films in 3D visualization.

**Figure 7:**
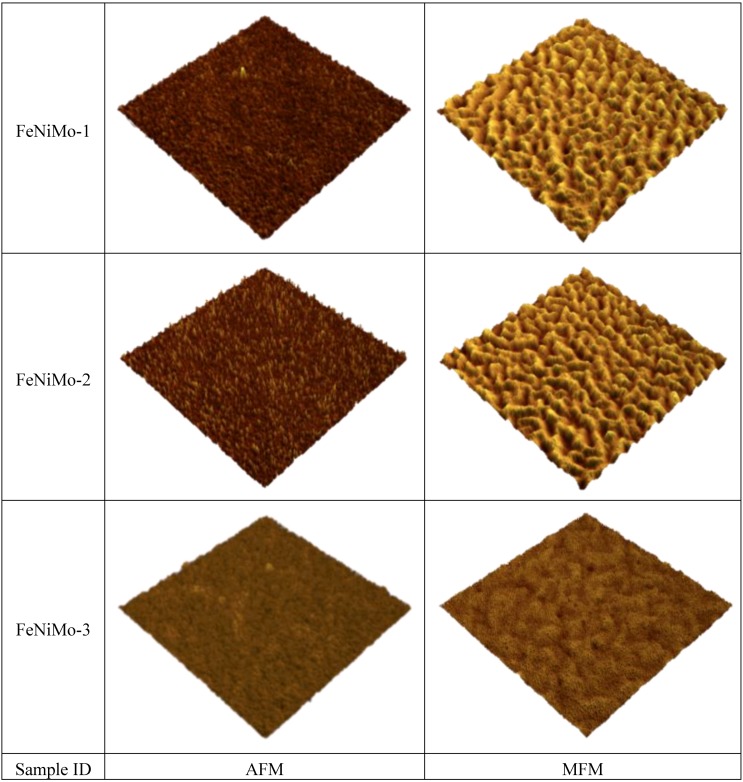
AFM and MFM images (size: 3 µm × 3 µm) of FeNiMo thin films in 3D visualization.

**Figure 8:**
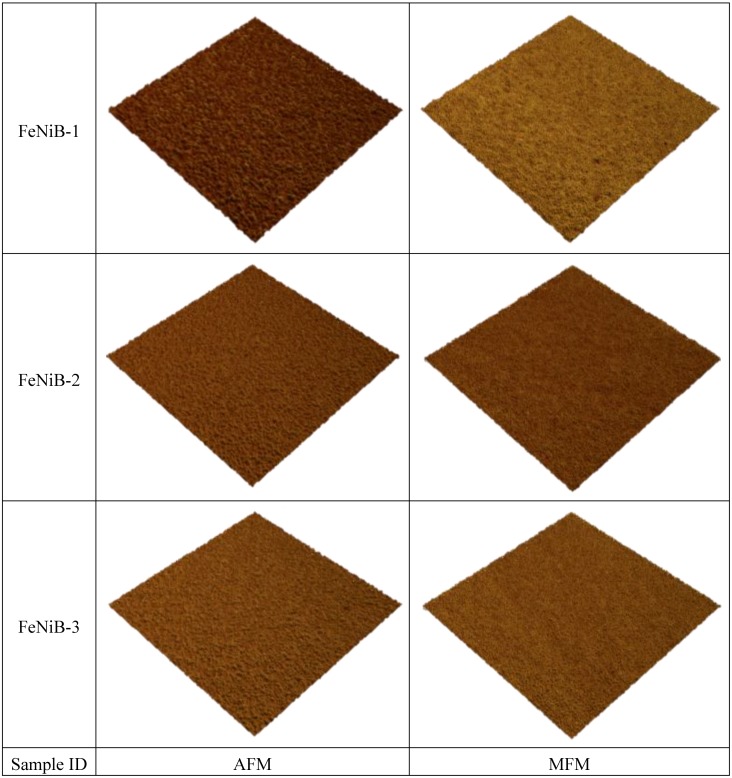
AFM and MFM images (size: 3 µm × 3 µm) of FeNiB thin films.

The out-of-plane magnetic properties were characterized by MFM. Figure 5 shows the 2D and 3D images of AFM and MFM for a FeNi film without any additives. It can be seen that the MFM images are significantly different from their counterpart AFM images both in 2D and 3D. Maze-like domain features exhibited in the MFM images are due to the strong perpendicular anisotropy in the film. For a better comparison and visualization, only 3D images of AFM and MFM are used for FeNi films with additives. Figure 6, Figure 7 and Figure 8 show the 3D images of FeNi films deposited with Al, Mo and B of various concentrations. The AFM images of FeNiAl showing a rough surface correspond well with the SEM images in Figure 1. The MFM images are significantly different from their AFM images, regardless of whether there are domain features in FeNiAl and FeNiMo or no domain features in FeNiB thin films. In the case of FeNiAl thin films, the out-of-plane magnetic components do not vanish with the increase of Al concentration, as can be seen in the Figure 6. It is sustained even at an Al concentration as high as 26.9 at%. Figure 7 show that the MFM images of FeNiMo change with the increase of Mo concentration; the visibility of domain features in FeNiMo decreases, and they nearly disappear when the concentration reaches 11.3 at%. In Figure 8, the MFM images of FeNiB films do not show the domain features. The AFM images reveal that these materials have a very fine and smooth surface topography, which agrees well with the SEM images in Figure 1g–i. These results show that depositing with B effectively breaks down the out-of-plane magnetic anisotropy of FeNi. In contrast, depositing with Al does not have this effect, and the magnetic anisotropy of FeNiMo films is found to depend on the concentration of Mo and is reduced at a higher concentration level, as also reported in [10].

Comparing FeNiB thin films with previously reported FeNiMoB thin films [10], we find that they share similarity in microstructure and magnetic properties. For instance, both types of materials have a very fine surface morphology, are amorphous in structure and have very soft ferromagnetic properties with coercivities as low as 40 A/m. In addition, they lack an out-of-plane magnetic anisotropy. Overall, the magnetic properties of both FeNiB and FeNiMoB films are very close to that of commercially available Metglas 2826 MB ribbons. However, the use of FeNiB will significantly reduce the complexity of the fabrication process towards integrated sensors.

## 4. Conclusions

FeNi thin films deposited with Mo, Al and B in various concentrations were studied in terms of microstructure (surface morphology, crystal structure) and magnetic properties (in-plane magnetization, out-of-plane domain structure). It was found that: (1) although depositing FeNi with Al made the thin films magnetically softer, it did not influence the crystal structures and did not break down the out-of-plane magnetic anisotropy; (2) depositing FeNi with B (i) changed the structure from crystalline to amorphous, (ii) reduced the coercivity to a very low value, and (iii) broke down the out-of-plane magnetic anisotropy; (3) depositing FeNi with Mo gradually changed the microstructure of FeNi and reduced the coercivity, as well as broke down the out-of-plane magnetic anisotropy with the increase of Mo concentration. The very smooth topography, amorphous nanostructure, the very low in-plane coercivity and the absence of out-of-plane magnetic anisotropy of FeNiB thin films were similar to the properties of the FeNiMoB thin films studied before and the Metglas 2826 MB ribbons. This indicated that B is an excellent additive element and that FeNiB thin films are promising substitutes for FeNiMoB films in microstructured sensors elements, where the deposition of only three elements (FeNiB) instead of four (FeNiMoB) would greatly simplify the manufacturing process. Future work will conduct the magnetostrictive constant measurement and fabricate the microscale devices for particular applications.
